# Identification of multimodal brain imaging association via a parameter decomposition based sparse multi-view canonical correlation analysis method

**DOI:** 10.1186/s12859-022-04669-z

**Published:** 2022-04-12

**Authors:** Jin Zhang, Huiai Wang, Ying Zhao, Lei Guo, Lei Du, Alzheimer’s Disease Neuroimaging Initiative

**Affiliations:** School of Automation, Northwestern Polytechnical University, Xi’an, China

**Keywords:** Sparse learning, Multi-view canonical correlation analysis, Parameter decomposition

## Abstract

**Background:**

With the development of noninvasive imaging technology, collecting different imaging measurements of the same brain has become more and more easy. These multimodal imaging data carry complementary information of the same brain, with both specific and shared information being intertwined. Within these multimodal data, it is essential to discriminate the specific information from the shared information since it is of benefit to comprehensively characterize brain diseases. While most existing methods are unqualified, in this paper, we propose a parameter decomposition based sparse multi-view canonical correlation analysis (PDSMCCA) method. PDSMCCA could identify both modality-shared and -specific information of multimodal data, leading to an in-depth understanding of complex pathology of brain disease.

**Results:**

Compared with the SMCCA method, our method obtains higher correlation coefficients and better canonical weights on both synthetic data and real neuroimaging data. This indicates that, coupled with modality-shared and -specific feature selection, PDSMCCA improves the multi-view association identification and shows meaningful feature selection capability with desirable interpretation.

**Conclusions:**

The novel PDSMCCA confirms that the parameter decomposition is a suitable strategy to identify both modality-shared and -specific imaging features. The multimodal association and the diverse information of multimodal imaging data enable us to better understand the brain disease such as Alzheimer’s disease.

## Background

Alzheimer’s Disease (AD) [1–5], the most common type of dementia, is a terrible neurodegenerative but its pathology is still unclear. And with the advance of imaging technologies, we can obtain multimodal imaging data of brain structure and function easily [6]. For example, the structural changes of the brain can be measured by structural magnetic resonance imaging (sMRI) scans, and the positron emission tomography (PET) scans can capture the brain activities such as the metabolic rate of glucose (FDG-PET) and amyloid depositions (AV45-PET) [7–10]. These different types of imaging data, including both modality-shared and -specific information, are collected simultaneously. As a result, it is essential to discriminate the modality-specific information from the modality-shared information, which could enable a better understanding of multimodal data and prompt reasonable multimodal brain imaging data integration [11–17].

The statistical pairwise correlation analysis has been widely used for medical image analysis. For example, researchers use both PET and functional magnetic resonance imaging(fMRI) data to study the relationship between brain and genes metabolism indicators [18]. With the deepening of research, researchers begin to use machine learning instead to focus on prediction tasks. However, they ignore the complex relationships in multimodal data. In contrast, exploring the correlation between multimodal brain imaging helps to reveal the pathogenesis of AD, thereby promotes the advancement of early diagnosis technology of the disease and the development of pharmaceutical research.

The existing correlation methods are mostly designed for two views [19, 20]. For instance, sparse canonical correlation analysis (SCCA) [21–27] has been widely used in brain imaging analysis. However, they cannot analyze multimodal imaging in a unified model. Although the multi-step strategy can be used to analyze the pairwise association between multiple modalities [2], it will inevitably cause the loss of potentially effective information. Thus these methods are sub-optimal. In order to analyze more than two modalities, SCCA can be directly and simply extended to multi-view paradigm [28] which has gained a lot of attentions. For example, based on sparse multiple/multi-view/multi-set canonical correlation analysis (SMCCA) [28, 29], researchers explored the association between multi-view data sets such as brain imaging data, genetic data and cognitive scores [30, 31]. However, SMCCA suffers from serious shortcomings which limit its application. First and foremost, SMCCA employs the $\ell _{1}$-norm, and thus cannot clearly report the modality-shared and -specific imaging features due to its overlook of the shared features of multiple modalities. In addition, the independent assumption of the in-set covariance of SMCCA makes the Pearson correlation coefficient break the range of $[-1,1]$, and there is no measure to avoid the additional risk caused by this assumption. According to [27], this independent assumption may not guarantee the convergence and consistency. Therefore, SMCCA is insufficient and inadequate in multimodal brain imaging analysis problem.

With above observations, to better identify the complex multi-way correlations among multimodal imaging data, we propose a novel sparse multi-view canonical correlation analysis (PDSMCCA) method based on the parameter decomposition. On the one hand, to improve interpretability, PDSMCCA contains two types of regularization($\ell _{1}$-norm and $\ell _{2,1}$-norm). The $\ell _{1}$-norm penalizes each imaging feature of each modality separately [32], and $\ell _{2,1}$-norm penalizes imaging features of multiple modalities jointly to obtain the modality-shared features [33, 34]. Using $\ell _{1}$-norm and $\ell _{2,1}$-norm together could offer a diverse feature selection. On the other hand, PDSMCCA decomposes the canonical weight into view-shared and -private components, which correspond to the modality-shared and -specific imaging features respectively. Owing to the decomposition strategy, PDSMCCA is able to obtain flexible imaging features. In addition, we relax the independent assumption of traditional SMCCA which treats the in-set covariance ${\mathbf {X}}^\top {\mathbf {X}}$ to be an identify [23]. Moreover, we introduce an efficient algorithm to solve the PDSMCCA model which converges to a local optimum. The results on synthetic data and real neuroimaging data show that, compared with the SMCCA method, our method obtains better or comparable canonical correlation coefficients (CCCs) and canonical weights. This indicates that our method is a powerful tool for multimodal brain imaging data association identification with diverse and desirable feature selection.

The contents of this article are arranged as follows. First, the SMCCA method is briefly introduced. Then, we describe the PDSMCCA in detail. Furthermore, we present the iterative optimization algorithm and prove its convergence, which is followed by experiments and results. Finally, the discussion and conclusion are provided.

## Experimental results

We use synthetic data and real data to evaluate the performance of our method and employ the state-of-the-art method (SMCCA) as the benchmark method. The experiment adopts the nested fivefold cross-validation and the grid search strategy to tune suitable $\lambda _B$ and $\lambda _S$, and the candidate parameter set is [0.01, 0.1, 1, 10, 100] which makes an appropriate feature selection since too large parameters and too small ones could incur undesirable features of interest. Besides, all methods are terminated when $\max |({\mathbf {b}}_k+{\mathbf {s}}_k)^{t+1}-({\mathbf {b}}_k+{\mathbf {s}}_k)^t| \le 10^{-5}$ is met. The canonical correlation coefficient (CCC) and the feature selection (heatmap) are utilized as the evaluation criteria. The CCC is defined as

1 $$\begin{aligned} {\mathbf{CCC}} = \frac{{\mathbf {v}}_i^\top {\mathbf {X}}_i^\top {\mathbf {X}}_j{\mathbf {v}}_j}{\sqrt{{\mathbf {v}}_i^\top {\mathbf {X}}_i^\top {\mathbf {X}}_i{\mathbf {v}}_i}\sqrt{{\mathbf {v}}_j^\top {\mathbf {X}}_j^\top {\mathbf {X}}_j{\mathbf {v}}_j}}, \end{aligned}$$

where ${\mathbf {X}}$ assumed to have been centered (zero mean), and ${\mathbf {v}}$ = ${\mathbf {b}} + {\mathbf {s}}$. For CCC, a larger score indicates a better performance of identifying the bi-associations among multiple modalities.

**Fig. 1:**
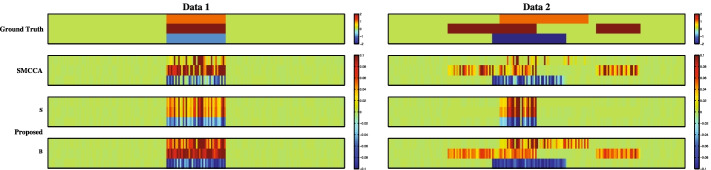
Comparison of canonical weights in terms of each method for two synthetic data sets. There are three modalities within each row and the meaning of the four rows are: (**1**) Ground Truth; (**2**) SMCCA; (**3**) PDSMCCA (S); (**4**) PDSMCCA (B)

### Results on synthetic data

In this simulation study, we use two synthetic data sets which contain different ground truth and noise intensity. We first generate three canonical weight vectors ${\mathbf {v}}_j \in {\mathbb {R}}^{200 \times 1}$ and a latent vector $\mu$ with unit norm. The data matrix ${\mathbf {X}}_k$ is generated by $({\mathbf {x}}_{i,j})_k \sim N(\mu _i{\mathbf {v}}_{j,k}, e \cdot {\mathbf {I}}_{200 \times 200})$, where *e* denotes the noise level.

- Data 1: $$n = 120$$, $${\mathbf {v}}_1=(\underbrace{0,...,0}_{80}, \underbrace{1,...,1}_{40}, \underbrace{0,...,0}_{80})^\top$$, $${\mathbf {v}}_2=(\underbrace{0,...,0}_{80}, \underbrace{2,...,2}_{40}, \underbrace{0,...,0}_{80})^\top$$, $${\mathbf {v}}_3=(\underbrace{0,...,0}_{80}, \underbrace{-1,...,-1}_{40}, \underbrace{0,...,0}_{80})^\top$$.
- Data 2: $$n = 120$$, $${\mathbf {v}}_1=(\underbrace{0,...,0}_{75}, \underbrace{1,...,1}_{60}, \underbrace{0,...,0}_{65})^\top$$, $${\mathbf {v}}_2=(\underbrace{0,...,0}_{40}, \underbrace{2,...,2}_{60}, \underbrace{0,...,0}_{40}, \underbrace{2,...,2}_{30}, \underbrace{0,...,0}_{30})^\top$$, $${\mathbf {v}}_3=(\underbrace{0,...,0}_{70}, \underbrace{-2,...,-2}_{50}, \underbrace{0,...,0}_{80})^\top$$.

In summary, we construct simulation data under different conditions to compare the proposed algorithm with the benchmark method.

**Table 1:** CCCs (mean ± SD) comparison on synthetic data

		Training CCCs		Testing CCCs	
		SMCCA	PDSMCCA	SMCCA	PDSMCCA
Data1					
${\mathbf {X}}_1-{\mathbf {X}}_2$		0.97 ± 0.00	**0.98** ± **0.00**	0.96 ± 0.03	**0.98** ± **0.01**
${\mathbf {X}}_1-{\mathbf {X}}_3$		0.95 ± 0.01	**0.98** ± **0.00**	0.93 ± 0.01	**0.97** ± **0.01**
${\mathbf {X}}_2-{\mathbf {X}}_3$		0.97 ± 0.00	**0.98** ± **0.00**	0.96 ± 0.01	**0.98** ± **0.01**
Data2					
${\mathbf {X}}_1-{\mathbf {X}}_2$		0.92 ± 0.03	**0.98** ± **0.00**	0.81 ± 0.09	**0.97** ± **0.01**
${\mathbf {X}}_1-{\mathbf {X}}_3$		0.91 ± 0.03	**0.98** ± **0.00**	0.82 ± 0.09	**0.98** ± **0.00**
${\mathbf {X}}_2-{\mathbf {X}}_3$		0.99 ± 0.03	**0.99** ± **0.00**	0.98 ± 0.00	**0.99** ± **0.00**

The highest values are shown in bold

Figure 1 shows the feature selection of the two methods on both synthetic data. It is worth noting that the intensity of the color reflects the relative importance of features. On the first data which only contains modality-shared features, both PDSMCCA and SMCCA can successfully identify these shared features. On the second data where both modality-shared and -specific features exist, SMCCA mixes these two types of features which is undesirable. On the contrary, PDSMCCA yields two types of features, including the modality-shared and -specific ones, which is more meaningful and practical. Table 1 presents the estimated canonical correlation coefficients between every two modalities. PDSMCCA obtains higher CCCs than SMCCA on both training and testing sets for two data sets. Therefore, PDSMCCA outperforms SMCCA in this simulation study.

### Results on real data

The brain imaging data were obtained from the Alzheimer’s Disease Neuroimaging Initiative (ADNI) database (https://adni.loni.usc.edu). and the primary goal of ADNI is to test whether serial magnetic resonance imaging (MRI), positron emission tomography (PET), other biological markers, and clinical and neuropsychological assessment can be combined to measure the progression of mild cognitive impairment (MCI) and early AD. For up-to-date information, see www.adni-info.org.

There were 755 samples including 281 ADs, 292 MCIs and 182 normal controls (NCs) non-Hispanic Caucasian participants. Three modalities of brain imaging data, including sMRI, FDG-PET and AV45-PET were used in this paper. FDG-PET and AV45-PET scans were co-registered to the standard MNI space. sMRI scans were processed with voxel-based morphometry (VBM) [35, 36] by the SPM software, and aligned to a T1-weighted template, then segmented to white matter (WM), gray matter (GM) and cerebrospinal fluid (CSF) maps, finally normalized to the same MNI space, and smoothed with an 8 ${\hbox {mm}}^3$ FWHM kernel. According to the automated anatomical labeling (AAL) atlas, we obtained 116 regions of interest (ROI) measurements. In order to eliminate the influence of baseline age, gender, habit, and education level, we used regression weights obtained from NC subjects to pre-adjust these imaging QTs. We aim to improve the interpretability of multimodal data for complex pathogenesis mechanisms, as well as select imaging QTs of interest.

**Fig. 2:**
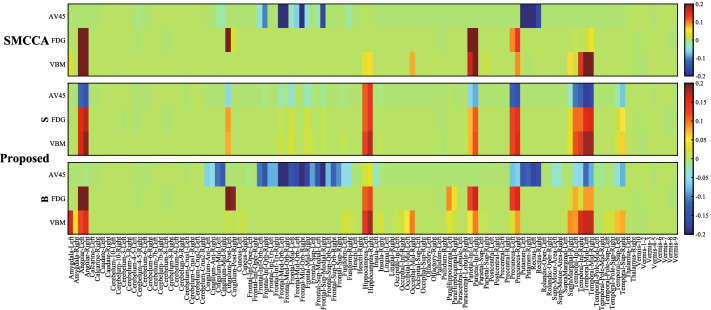
Canonical weights on real data. The top row belongs to SMCCA, and the remaining two rows correspond to the shared and specific results of our method. Within each panel, there are three rows corresponding to three types of imaging QTs, i.e. AV45, FDG and VBM

Figure 2 shows the feature selection results on real neuroimaging data. According to the intensity of the color, we can determine the relative importance of features. It is clear that PDSMCCA identifies more diverse imaging QTs than SMCCA. For the modality-shared features conveyed by ${\mathbf {S}}$, PDSMCCA identifies the left and right hippocampus [4, 37], the left and right middle temporal [38], the left and right precuneus as the most relevant shared ROIs. Besides, PDSMCCA also identifies the modality-specific features which is shown in weight ${\mathbf {B}}$. It is clear that the left and right medial orbitofrontal [9] are relevant only in AV45 scans [20, 39]. Meanwhile, the left post cingulum is relevant in FDG scans, and both the left and right hippocampus are relevant in sMRI scans. In contrast, SMCCA misses the brain regions shared by multiple modalities, since it cannot obtain the diverse feature selection results. It mixes both modality-shared features and modality-specific ones which is insufficient in real applications. We also present the CCCs of both methods in Table 2. Our method obtains better CCCs than SMCCA, which indicates that our method can identify stronger bi-multivariate associations. In summary, PDSMCCA holds the capability to identify the multi-way correlations between multiple modalities of data, and can identify more meaningful features.

**Fig. 3:**
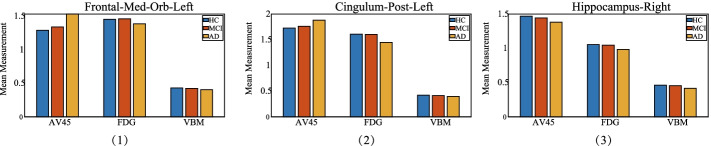
The top selected imaging QT of each modality and their distribution among distinct diagnostic groups. (**1**) The Frontal-Med-Orb-Left. (**2**) The Cingulum-Post-Left. (**3**) The Hippocampus-Right

To further show the meaning of these selected imaging QTs, the ANOVA and population stratification analysis were conducted. The one-way ANOVA results showed that the top selected imaging QTs reached the level of significance (*p* < 0.01). This indicated imaging QTs were significantly related to the diagnosis. Moreover, in order to verify the biological effects of the selected imaging QTs. We further analyzed the prominent imaging QT of each modality, which were Frontal-Med-Orb-Left in AV45 [40], Cingulum-Post-Left [41] in FDG and Hippocampus-Right [37] in VBM. Since there were three diagnostic groups, we decided to investigate whether they were significantly different among different groups. Figure 3 showed that Frontal-Med-Orb-Left and Cingulum-Post-Left exhibited significant changes in FDG and AV45 which was consistent with the decline of metabolic rates of cerebral glucose and the variety of extracellular amyloid deposition. Besides, the Hippocampus-Right showed consistent patterns that decreased measurement were observed in all modalities. This may be attributed to its high correlation to AD. In summary, benefiting from the parameter decomposition strategy, our proposed method can obtain interesting and meaningful biomarkers in multimodal brain imaging analysis.

**Table 2:** CCCs (mean ± SD) estimated between three types of imaging QTs

		AV45-FDG	AV45-VBM	FDG-VBM
Training				
SMCCA		0.33 ± 0.01	0.28 ± 0.02	0.50 ± 0.02
PDSMCCA		**0.35** ± **0.01**	**0.31** ± **0.01**	0.49 ± 0.01
Testing				
SMCCA		0.32 ± 0.01	0.24 ± 0.01	0.49 ± 0.02
PDSMCCA		**0.33** ± **0.01**	**0.28** ± **0.01**	0.48 ± 0.01

The highest values are shown in bold

## Discussion

Generally, different techniques yield different measurements of the same brain, and could carry shared and specific information simultaneously. In this paper, PDSMCCA is proposed to explore the multi-way relationship among multiple brain imaging modalities, and it can identify both modality-shared and -specific imaging features through the parameter decomposition technology. Importantly, this decomposition technology is flexible via balancing between two contradictory constraints ($\ell _{1}$-norm and $\ell _{2,1}$-norm), and thus assures a better performance [42]. This improves the interpretability of traditional SMCCA method. Of note, similar to SMCCA, PDSMCCA is also unsupervised which could be a limitation. The future work is to incorporate the diagnostic labels into the PDSMCCA model, and build a supervised method to better mine the brain imaging association with selecting relevant imaging features.

## Conclusion

To improve the interpretability of multimodal data for complex pathogenesis mechanisms, we proposed a novel sparse multi-view canonical correlation analysis method (PDSMCCA) based on parameter decomposition. In our model, the canonical weights were decomposed into modality-shared and modality-specific components, resulting in a flexible and meaningful interpretability. We also introduced an efficient optimization algorithm to solve PDSMCCA, and proved the convergence. The results on both synthetic and real neuroimaging data showed that compared with SMCCA, PDSMCCA accurately selected the modality-shared and -specific features, and obtained higher or comparable correlation coefficients. The diverse feature selection might provide a new insight for revealing AD pathology.

## Method

In this paper, italic letters indicate scalars, boldface lowercase letters and boldface capitals represents column vectors and matrices respectively. Specifically, the *i-*th row and *j-*th column of ${\mathbf {V}}$ is denoted as ${\mathbf {v}}^i$ and ${\mathbf {v}}_j$.  $\Vert {{\mathbf {V}}}\Vert _{2,1} = \sum _i \Vert {{\mathbf {v}}^{i}}\Vert _2$ is the $\ell _{2,1}$-norm. In addition,  $\Vert {{\mathbf {V}}}\Vert _{1,1}$ denotes the element-wise $\ell _1$-norm of ${\mathbf {V}}$, i.e., $\Vert {{\mathbf {V}}}\Vert _{1,1}=\sum _j \Vert {{\mathbf {v}}_{j}}\Vert _1=\sum _i \Vert {{\mathbf {v}}^{i}}\Vert _1=\sum _i\sum _j |{v_{ij}}|$.

### SMCCA

SMCCA extends the conventional two-view SCCA model to multi-view oriented, which can handle the association identification among multiple data sets. Generally, the definition of SMCCA is as follows:

2 $$\begin{aligned} \min _{{\mathbf {v}}_k}&\sum _{1 \le k, j \le K} \left( -{\mathbf {v}}_k^\top {\mathbf {X}}_k^\top {\mathbf {X}}_j {\mathbf {v}}_j + \lambda \Vert {{\mathbf {v}}_k}\Vert _1 \right) \\&{\text{ s.t. }} ~~ {\Vert {{\mathbf {v}}_k}\Vert _2^2}=1~~(k = 1,\ldots , K). \end{aligned}$$

According to [43, 44], (2) can be rewritten as a multivariate multiple regression model.

3 $$\begin{aligned} \min _{{\mathbf {v}}_k} \sum _{1 \le k, j \le K}&\left( \frac{1}{2}\Vert {{\mathbf {X}}_k {\mathbf {v}}_k - {\mathbf {X}}_j{\mathbf {v}}_j}\Vert _2^2 + \lambda \Vert {{\mathbf {v}}_k}\Vert _1 \right) \\ {\text{ s.t. }} ~~&{\Vert {{\mathbf {v}}_k}\Vert _2^2}=1~~(k =1,\ldots , K), \end{aligned}$$

where ${\mathbf {X}}_k \in {\mathbb {R}}^{n \times p}(k =1,\ldots , K)$ represents the *k-*th modality of imaging data with *n* samples and *p* imaging quantitative traits (QTs) and *K* is the number of imaging modalities. ${\mathbf {v}}_k \in {\mathbb {R}}^{p \times 1}$ represents the canonical weight corresponding to the *k-*th modality, and ${\mathbf {V}}=[{\mathbf {v}}_{1},\ldots ,{\mathbf {v}}_{K}]$. These weights yielded by SMCCA show the importance of each imaging feature in associating multiple brain imaging modalities. However, SMCCA supposes ${\mathbf {X}}_k^\top {\mathbf {X}}_k = {\mathbf {I}}$ which weakens the performance of the model [23]. What’s worse, the modality-shared imaging features mix up with those modality-specific ones, resulting in poor interpretability.

### PDSMCCA

In order to better identify the relationship between multimodal brain imaging data and overcome the drawbacks of SMCCA, we propose a novel SMCCA (PDSMCCA) model. PDSMCCA is defined as follows:

4 $$\begin{aligned} \min _{{\mathbf {b}}_k, ~ {\mathbf {s}}_k}&\sum _{1\le {k}, ~ {j}\le K}^K {\frac{1}{2} \Vert {{\mathbf {X}}_k({\mathbf {b}}_k+{\mathbf {s}}_k)-{\mathbf {X}}_j({\mathbf {b}}_j+{\mathbf {s}}_j)}\Vert _2^2}+ \lambda _B \Vert {{\mathbf {B}}}\Vert _{1,1} \\ +&\lambda _S \Vert {{\mathbf {S}}}\Vert _{2,1},~~ {\text{ s.t. }}~\Vert {{\mathbf {X}}_k({\mathbf {b}}_k+{\mathbf {s}}_k)}\Vert _2^2=1~ (k= 1,\ldots , K),\\ \end{aligned}$$

where $\lambda _B$ and $\lambda _S$ are two nonnegative tuning parameters, and ${\mathbf {V=B+S}}$. The decomposition of ${\mathbf {V}}$ is interesting and meaningful.

Specifically, by using different regularization functions for ${\mathbf {B}}$ and ${\mathbf {S}}$, we can enable them to select different types of features, e.g. the modality-shared and -specific features. In this paper, we impose the $\ell _{2,1}$-norm [33] on ${\mathbf {S}}$ to select the shared features across multiple modalities, and this penalty is defined as $\Vert {{\mathbf {S}}}\Vert _{2,1} = \sum _i \Vert {{\mathbf {s}}^{i}}\Vert _2$. In addition, we use the $\ell _{1}$-norm for an imaging QT across all imaging modalities. This might identify features that can only be recognized under certain technologies. And the penalty is defined as $\Vert {{\mathbf {B}}}\Vert _{1,1}=\sum _j \Vert {{\mathbf {b}}_{j}}\Vert _1=\sum _i \Vert {{\mathbf {b}}^{i}}\Vert _1=\sum _i\sum _j |{b_{ij}}|$.

The merits of PDSMCCA are as follows. First of all, our model directly calculates the multi-way association among multiple data modalities, which holds a powerful modeling capability. Besides, we use $\ell _{1}$-norm to identify related QTs that may only change in a single imaging modality, and use $\ell _{2,1}$-norm to identify related imaging QTs that change together due to the co-varying effects of AD, which demonstrates a diverse and desirable feature selection capability. Most importantly, attributing to the parameter decomposition and diverse regularization, the modality-shared features and modality-specific features can be obtained in a unified model, which could provide a better interpretation for biomedical studies.

### The optimization algorithm

According to Lemma 2.2 in [45], the optimum ${\mathbf {b}}_k$ and ${\mathbf {s}}_k$ can be obtained by ${\mathbf {b}}_k^* = \frac{\hat{{\mathbf {b}}_k}}{\Vert {{{{{\mathbf {X}}_k}({\mathbf {b}}_k+{\mathbf {s}}_k)}}}\Vert _2}$ and ${\mathbf {s}}_k^* = \frac{\hat{{\mathbf {s}}_k}}{\Vert {{{{{\mathbf {X}}_k}({\mathbf {b}}_k+{\mathbf {s}}_k)}}}\Vert _2}$ respectively. Further, $\hat{{\mathbf {b}}_k}$ and $\hat{{\mathbf {s}}_k}$ are solutions to the following objective,

5 $$\begin{aligned} \min _{{\mathbf {b}}_k,{\mathbf {s}}_k} \sum _{1\le {k}, ~ {j}\le K}^K { \frac{1}{2} \Vert {{\mathbf {X}}_k({\mathbf {b}}_k+{\mathbf {s}}_k)-{\mathbf {X}}_j({\mathbf {b}}_j+{\mathbf {s}}_j)}\Vert _2^2}+ \lambda _B \Vert {{\mathbf {B}}}\Vert _{1,1} +\lambda _S \Vert {{\mathbf {S}}}\Vert _{2,1}. \end{aligned}$$

Equation (5) is a typical bi-convex function, and we can use the alternating convex search (ACS) method [46] to solve this objective. That is, we update one variable and fix all the remaining ones at each step. Since ${||{{\mathbf {X}}_k}({\mathbf {b}}_k+{\mathbf {s}}_k)||_2^2}=1$, (5) is processed as follows:

6 $$\begin{aligned} \min _{{\mathbf {b}}_k,{\mathbf {s}}_k}&\sum _{1 \le k, j \le K}^K {\frac{1}{2}||{\mathbf {X}}_k({\mathbf {b}}_k+{\mathbf {s}}_k)-{\mathbf {X}}_j({\mathbf {b}}_j+{\mathbf {s}}_j)||_2^2}\\&-\frac{1}{4}\Vert {{\mathbf {X}}_k({\mathbf {b}}_k+{\mathbf {s}}_k)}\Vert _2^2 + \frac{1}{2}\Vert {{\mathbf {X}}_j({\mathbf {b}}_j+{\mathbf {s}}_j)}\Vert _2^2\\&+{\lambda _B}{\mathbf {||B||}_{1,1}} +{\lambda _S}{\mathbf {||S||}_{2,1}},\\ \end{aligned}$$

according to inequality ${{\frac{1}{4}\Vert {{\mathbf {X}}_k({\mathbf {b}}_k+{\mathbf {s}}_k)}\Vert _2^2}}\le {\frac{1}{2}{\Vert {{\mathbf {X}}_k{\mathbf {b}}_k}\Vert _2^2}}+{\frac{1}{2}{\Vert {{\mathbf {X}}_k{\mathbf {s}}_k}\Vert _2^2}}$, we equivalently have the following objective with respect to ${\mathbf {b}}_k$ and ${\mathbf {s}}_k$,

7 $$\begin{aligned} \min _{{\mathbf {b}}_k,{\mathbf {s}}_k}&\sum _{1 \le k, j \le K}^K \frac{1}{2}\Vert {{\mathbf {X}}_k{\mathbf {b}}_k-{\mathbf {X}}_j({\mathbf {b}}_j+{\mathbf {s}}_j)}\Vert _2^2 \\&\quad +\frac{1}{2}\Vert {{\mathbf {X}}_k{\mathbf {s}}_k-{\mathbf {X}}_j({\mathbf {b}}_j+{\mathbf {s}}_j)}\Vert _2^2 + \lambda _B \Vert {{\mathbf {B}}}\Vert _{1,1} + \lambda _S \Vert {{\mathbf {S}}}\Vert _{2,1}. \end{aligned}$$

Equation (7) is convex in ${\mathbf {b}}_k$ when fixing ${\mathbf {s}}_k$ as constants.

8 $$\begin{aligned} \min _{{\mathbf {b}}_k}&\sum _{1 \le k, j \le K}^K{\frac{1}{2}\Vert {{\mathbf {X}}_k{\mathbf {b}}_k-{\mathbf {X}}_j({\mathbf {b}}_j+{\mathbf {s}}_j)}\Vert _2^2} + \lambda _B \Vert {{\mathbf {b}}_k}\Vert _1, \end{aligned}$$

Then based on the ACS strategy, we take the derivative with respect to each ${\mathbf {b}}_k$, and letting it be zero, we obtain

9 $$\begin{aligned} {\mathbf {b}}_k = ({\lambda _B}{\mathbf {D}}_b+{(K-1)} {\mathbf {X}}_k^T {\mathbf {X}}_k)^{-1} \sum _{1 \le k, j \le K}^K {\mathbf {X}}_k^T{\mathbf {X}}_j({\mathbf {b}}_j+{\mathbf {s}}_j), \end{aligned}$$

where ${\mathbf {D}}_b$ is a diagonal matrix with the *i*th diagonal element being $\frac{1}{|{{\mathbf {b}}_{ik}}|}$.

Similarly, the optimal ${\mathbf {s}}_k$ can be obtained by solving (10)

10 $$\begin{aligned} \min _{{\mathbf {s}}_k}&\sum _{1 \le k, j \le K}^K{\frac{1}{2}\Vert {{\mathbf {X}}_k{\mathbf {s}}_k-{\mathbf {X}}_j({\mathbf {b}}_j+{\mathbf {s}}_j)}\Vert _2^2} + \lambda _S \Vert {{\mathbf {S}}}\Vert _{2,1}. \end{aligned}$$

then we have the closed-from updating rule for each ${\mathbf {s}}_k$,

11 $$\begin{aligned} {\mathbf {s}}_k = ({\lambda _S}{\mathbf {D}}_s+{(K-1)}{\mathbf {X}}_k^T{\mathbf {X}}_k)^{-1} \sum _{1 \le k, j \le K}^K{{\mathbf {X}}_k^T{\mathbf {X}}_j({\mathbf {b}}_j+{\mathbf {s}}_j)}, \end{aligned}$$

where ${\mathbf {D}}_s$ is a diagonal matrix, and its *i*th diagonal element is $\frac{1}{\Vert {{\mathbf {s}}^{i}}\Vert _{2}}$ ($i=1,\ldots ,p$).

Once every ${\mathbf {b}}_k$ and ${\mathbf {s}}_k$ is attained, ${\mathbf {B}}$ and ${\mathbf {S}}$ can be attained as well. Finally, we present the pseudo-code in Algorithm 1. The input of PDSMCCA is the neuroimaging quantitative trait data from multiple modalities, and the output is the canonical weight (absolute value) showing the relative importance of each imaging feature. Step 1 initializes ${\mathbf {B}}$ and ${\mathbf {S}}$. Step 3 to 6 are iteration procedure to seek the final solutions.

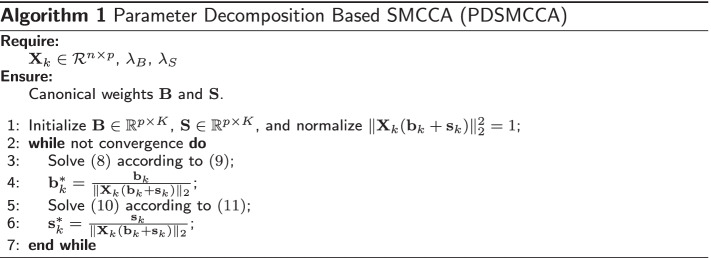



### Convergence analysis

Theorem 1 will prove that Algorithm 1 converge to a local optimum.

#### **Theorem 1**

*The value of* (4) *keeps decreasing througout the iteration of Algorithm 1.*

We use $\left\{ {\mathbf {b}}_k^{(t)}, {\mathbf {s}}_k^{(t)}\right\}$ to represent the estimate of $\left\{ {\mathbf {b}}_k, {\mathbf {s}}_k\right\}$ in the *t*th iteration. Next, we will prove that the value of (8) is continuously decreasing when solving ${\mathbf {b}}_k$. To facilitate understanding, we denote the objective of (8) as $F\left( {\mathbf {b}}_k\right)$:

12 $$\begin{aligned} F\left( {\mathbf {b}}_k\right) =\sum _{1 \le k, j \le K}^K{\frac{1}{2}\Vert {{\mathbf {X}}_k{\mathbf {b}}_k-{\mathbf {X}}_j({\mathbf {b}}_j^{(t)}+{\mathbf {s}}_j^{(t)})}\Vert _2^2} + \lambda _B \Vert {{\mathbf {b}}_k}\Vert _1. \end{aligned}$$

Then we define

13 $$\begin{aligned} G\left( {\mathbf {b}}_k\right)&=\sum _{1 \le k, j \le K}^K \frac{1}{2}\Vert {{\mathbf {X}}_k{\mathbf {b}}_k-{\mathbf {X}}_j({\mathbf {b}}_j^{(t)}+{\mathbf {s}}_j^{(t)})}\Vert _2^2+ \lambda _B \sum _{i=1}^p \left( \frac{b_{ki}^2}{2|{b_{ki}^{(t)}}|} +\frac{|{b_{ki}^{(t)}}|}{2}\right) \\&=\sum _{1 \le k, j \le K}^K{\frac{1}{2}\Vert {{\mathbf {X}}_k{\mathbf {b}}_k-{\mathbf {X}}_j({\mathbf {b}}_j^{(t)}+{\mathbf {s}}_j^{(t)})}\Vert _2^2} + \lambda _B\left( \frac{1}{2}{\mathbf {b}}_k^\top {\mathbf {D}}_b {\mathbf {b}}_k + \frac{1}{2}{{\mathbf {b}}_k^{(t)}}^\top {\mathbf {D}}_b {\mathbf {b}}_k^{(t)}\right) , \end{aligned}$$

where ${\mathbf {D}}_b$ is defined in (9), and (14) can be easily proved. It is obvious that $G\left( {\mathbf {b}}_k\right)$ is a convex quadratic function that satisfies

14 $$\begin{aligned} G\left( {\mathbf {b}}_k^{(t)}\right) = F\left( {\mathbf {b}}_k^{(t)}\right) ,~~ G\left( {\mathbf {b}}_k\right) \ge F\left( {\mathbf {b}}_k\right) , \; \forall {\mathbf {b}}_k \in {\mathbb {R}}^p. \end{aligned}$$

Since the estimate of ${\mathbf {b}}_k$ at the next iteration $t+$1, expressed in (8) and denoted as ${\mathbf {b}}_k^{(t+1)}$, is the minimizer of $G\left( {\mathbf {b}}_k\right)$, we have

15 $$\begin{aligned} G\left( {\mathbf {b}}_k^{(t+1)}\right) \le G\left( {\mathbf {b}}_k^{(t)}\right) . \end{aligned}$$

Putting (13)–(15) together, we have

16 $$\begin{aligned} F\left( {\mathbf {b}}_k^{(t+1)}\right) \le G\left( {\mathbf {b}}_k^{(t+1)}\right) \le G\left( {\mathbf {b}}_k^{(t)}\right) = F\left( {\mathbf {b}}_k^{(t)}\right) . \end{aligned}$$

This formula shows that the objective decreases by fixing ${\mathbf {s}}_k$, which guarantees the convergence. And after the rescaling, the conclusion is still valid. Thus, for ${\mathbf {s}}_k$, we can get the same conclusion in the same way. By denoting the objective as ${\mathcal {L}}({\mathbf {b}}_k,{\mathbf {s}}_k)$, then according to the conclusions above, we have

17 $$\begin{aligned} \begin{gathered} {\mathcal {L}}({\mathbf {b}}_k^{(t+1)},{\mathbf {s}}_k^{(t+1)}) \le {\mathcal {L}}({\mathbf {b}}_k^{(t+1)},{\mathbf {s}}_k^{(t)}) \le {\mathcal {L}}({\mathbf {b}}_k^{(t)},{\mathbf {s}}_k^{(t)}), \end{gathered} \end{aligned}$$

We further know ${\mathcal {L}}({\mathbf {b}}_k,{\mathbf {s}}_k )$ is lower bounded by zero. Therefore, we combine (16)–(17), Algorithm 1 will converge to the optimum.

## Data Availability

Data used in this study are publicly available at the ADNI database (www.adni-info.org). They are from the ADNI1, ADNI GO and ADNI2 standard data sets that are considered in experiments with the MRI and PET data sets.

## Abbreviations

CCC: Canonical correlation coefficient
PDSMCCA: Parameter decomposition based sparse multi-view canonical correlation analysis
AD: Alzheimer’s Disease
ADNI: Alzheimer’s Disease Neuroimaging Initiative
ACS: Alternating convex search
MRI: Magnetic resonance imaging
MCI: Mild cognitive impairment
sMRI: Structural magnetic resonance imaging
FDG: Fluorodeoxyglucose
QTs: Quantitative traits
ROI: Regions of interest
AAL: Automated anatomical labeling
CSF: Cerebrospinal fluid
GM: Gray matter
WM: White matter
SCCA: Sparse canonical correlation analysis
SMCCA: Sparse multiple/multi-view/multi-set canonical correlation analysis
NC: Normal controls
AV45: 18-F florbetapir PET
VBM: Voxel-based morphometry
PET: Positron emission tomography
fMRI: Functional magnetic resonance imaging
